# Portable 3D-printed hand orthosis with spatial stiffness distribution personalized for assisting grasping in daily living

**DOI:** 10.3389/fbioe.2023.895745

**Published:** 2023-02-02

**Authors:** Chan Beom Park, Hyung-Soon Park

**Affiliations:** Department of Mechanical Engineering, Korea Advanced Institute of Science and Technology (KAIST), Daejeon, South Korea

**Keywords:** hand orthosis, personalized, portable, spatial stiffness distribution, strengthful, stroke

## Abstract

Stroke survivors having limited finger coordination require an active hand orthosis to assist them with grasping tasks for daily activities. The orthosis should be portable for constant use; however, portability imposes constraints on the number, size, and weight of the actuators, which increase the difficulty of the design process. Therefore, a tradeoff exists between portability and the assistive force. In this study, a personalized spatial stiffness distribution design is presented for a portable and strengthful hand orthosis. The spatial stiffness distribution of the orthosis was optimized based on measurements of individual hand parameters to satisfy the functional requirements of achieving sufficient grip aperture in the pre-grasping phase and minimal assistive force in the grasping phase. Ten stroke survivors were recruited to evaluate the system. Sufficient grip aperture and high grip strength-to-weight ratio were achieved by the orthosis *via* a single motor. Moreover, the orthosis significantly restored the range of motion and improved the performance of daily activities. The proposed spatial stiffness distribution can suggest a design solution to make strengthful hand orthoses with reduced weight.

## 1 Introduction

Stroke is a cerebrovascular event that occurs in 13 million people worldwide every year (Lindsay et al., 2019). Often, it leads to neurological deficits such as spasticity, muscle weakness, and muscle co-contraction in the upper extremities, which negatively affects activities of daily living (ADL) (Chae et al., 2002; Kuo and Hu, 2018). Early in the post-stroke stage, intensive and task-specific training is implemented with the help of therapists and rehabilitation devices to restore impaired motor functions (Bayona et al., 2005; Waddell et al., 2014). However, ∼65% of stroke survivors in the chronic stage cannot use their affected hands to perform ADL owing to challenges in hand function recovery (Dobkin, 2005; Wang et al., 2019). Portable hand orthoses, which can assist with finger movements according to user intentions, are necessary to aid stroke survivors with grasping tasks for ADL (Peters et al., 2017).

For stroke survivors, the limited finger coordination because of spasticity and muscle weakness is the primary obstacle for performing grasping tasks with the affected hand (Kamper et al., 2006; Kuo and Hu, 2018). In particular, flexor spasticity and extensor muscle weakness limit the range of motion (ROM) of finger joints (Hoffmann et al., 2016; Kuo and Hu, 2018). Thus, the orthosis should support both finger flexion and extension. Including actuators, a portable orthosis should weigh <500 g because of its constant use and for easy donning/doffing (Nycz et al., 2016; Ates et al., 2017; Lambelet et al., 2020). Moreover, it should be able to counteract spasticity in the pre-grasping phase to ensure a sufficient grip aperture, and it should generate a grip strength above 15 N (Yurkewich et al., 2019).

Many types of hand orthoses have been developed. Most existing hand orthoses include multiple actuators to assist dexterous finger movements for rehabilitation purposes. The hand orthoses comprise exoskeletons (Ryu et al., 2008; Chiri et al., 2009; Ho et al., 2011; Ates et al., 2015), tendon-driven gloves (Wege and Zimmermann, 2007; Ueki et al., 2012; Aiple and Schiele, 2013; Borboni et al., 2016; Xiloyannis et al., 2016; Kim and Park, 2018; Burns and Vinjamuri, 2020; Alicea et al., 2021), and inflatable robotic gloves (Cappello et al., 2018; Nuckols et al., 2020). The exoskeletal orthoses can assist fingers with accurately controlled joint movements; however, they inherently suffer from poor portability due to their heavy weight. The soft orthoses considerably reduce applied weight on the hand using gloves made from lightweight silicone or fabric; however, they are less convenient to be used in daily living due to multiple actuators with long tendon sheaths and tubes required to transfer assistive force from the actuators to the gloves.

Several portable hand orthoses have been developed by reducing the number of actuators in the soft orthoses for assistive purposes in daily living (In et al., 2015; Polygerinos et al., 2015; Thielbar et al., 2016; Popov et al., 2017; Yap et al., 2018; Yurkewich et al., 2019; 2020). Some portable orthoses (Popov et al., 2017; Yurkewich et al., 2019; 2020) even do not require any long tendon sheaths and tubes for convenient use. Although multiple portable hand orthoses have been developed for stroke survivors, they have not been quantitatively evaluated for their effectiveness at assisting with grasping tasks for ADL due to an issue regarding grip strength. It is because the assistive force of portable hand orthoses is limited by the allowable number, size, and weight of actuators, which inhibits finger extension and grip strength (Yurkewich et al., 2019; 2020). Many researchers have developed portable orthoses of which actuation modules are remotely mounted on the body such as on the waist (Polygerinos et al., 2015; Thielbar et al., 2016; Yap et al., 2018) to reduce the external weight on the hand; however, heavy actuators are still associated with poor usability.

An elastic structure can be used for portable orthoses (Arata et al., 2013; Nycz et al., 2016) to ensure passive finger extension, thus reducing the required number of actuators for grasping assistance. The stiffness of the structure significantly affects grasping performance for each individual; the low stiffness would result in limited grip aperture in the pre-grasping phase by spastic finger joints while the high stiffness would lead to limited grip strength in the grasping phase by requiring unnecessarily high assistive force for flexion. Thus, it is desirable to personalize the spatial stiffness distribution of the structure based on individual degrees of spasticity (Park et al., 2016) to achieve sufficient grip aperture and grip strength with actuators constrained by number, size, and weight. However, there are limited design guidelines to determine the spatial stiffness distribution of hand orthoses for each individual.

**FIGURE 1:**
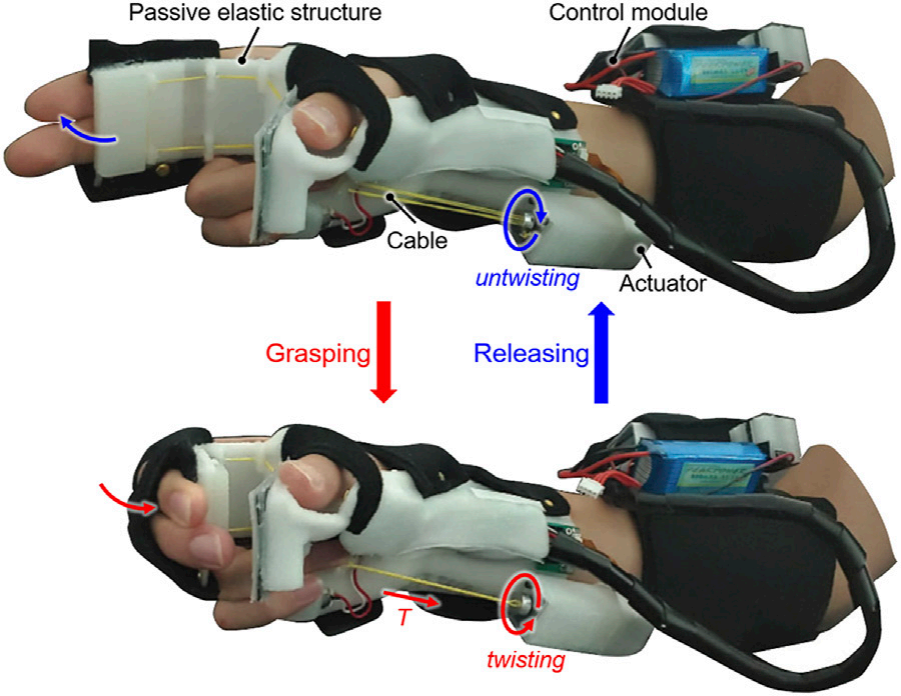
The active hand orthosis made by the spatial stiffness distribution design for assisting with grasping tasks during activities of daily living (ADL). The entire orthosis, including the single actuator, was designed to fit compactly on the hand and forearm.

This study presents a personalized spatial stiffness distribution design for a portable and strengthful hand orthosis to assist with grasping tasks (Figure 1). The spatial stiffness distribution is designed as per individuals to achieve a sufficient grip aperture against spasticity in the pre-grasping phase and a minimal assistive force in the grasping phase. Because the degree of spasticity is unique to each stroke survivor, the spatial stiffness distribution is optimized based on measurements of individual hand parameters. The orthosis having the personalized spatial stiffness distribution is made using a 3D printer, and it was evaluated by recruiting chronic stroke survivors with impaired hand functionality. By using only a single small (10 ∅ ) motor, the orthosis can assist with grasping tasks by generating a sufficient grip aperture and high grip strength. The rest of this study is organized as follows. Section 2 presents an overview of the orthosis design and describes the procedure for optimizing the stiffness of the orthosis. Section 2 also presents the experimental evaluation. Section 3 presents the experimental results. Section 4 discusses the significance and implications of the results in this study and concludes the paper.

## 2 Materials and methods

### 2.1 Overall design

The specifications of the orthosis made by the spatial stiffness distribution design are determined as follows based on the results of previous studies and interviews with clinicians.

1) Portability: The orthosis including the actuators should weigh <500 g (Nycz et al., 2016; Ates et al., 2017; Lambelet et al., 2020) because it will be in constant use throughout the day. Thus, the number of actuators should be minimized.
2) Grasping assistance: The orthosis should assist with the bidirectional movement of the fingers to aid with grasping tasks. The target grip aperture and minimal required grip strength are set to 80 mm (>70 mm (Feix et al., 2014)) and 15 N (Yurkewich et al., 2019), respectively.
3) Comfort and safety: The orthosis should not induce pain, hyperextension, and skin damage during use. The application of a compressive force to finger joints must be avoided when it is assisting with finger movement.

The number of grasp types that the orthosis can assist with is limited because the underactuation of the orthosis is inevitable for portability. Because tripod grasp achieved by three fingers (i.e., the thumb, index finger, and middle finger) was similar with the grasping posture achieved by one of the postural synergies required for a tool use (Santello et al., 1988), it was considered that common objects can be grasped by the tripod grasp. Thus, the tripod grasp is selected for emulation using a single motor.

**FIGURE 2:**
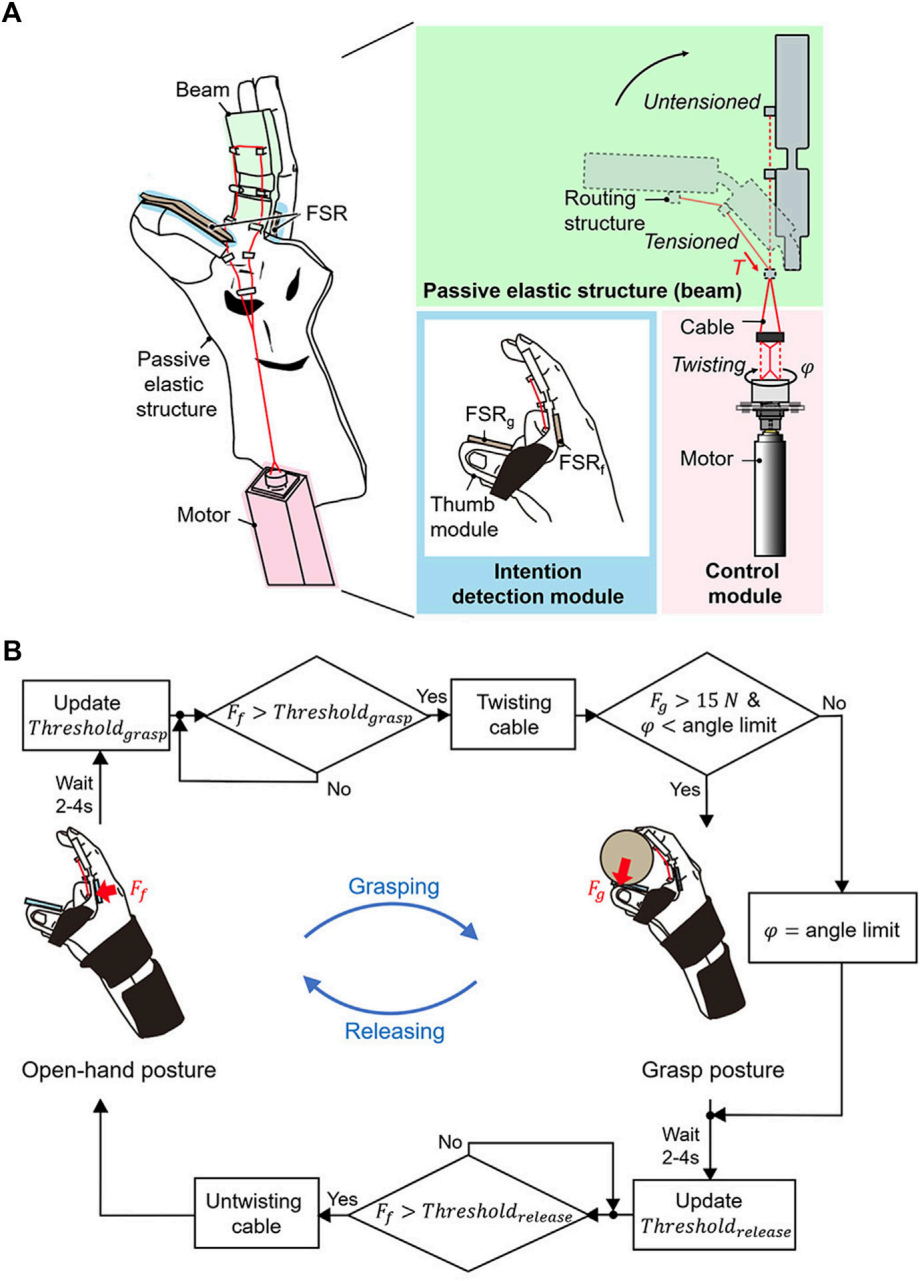
Design overview of the active hand orthosis. **(A)** The orthosis consists of a passive elastic structure for passive extension, a control module for active flexion, and an intention detection module. The rotation angle ( φ ) of the motor is adjusted to twist the cable, which generates the assistive force (*T*). The assistive force is applied to routing structures of the beam. Two force-sensing resistors (FSR_g_, FSR_f_) are used as the intention detection module. **(B)** Working principle of the orthosis based on user intention. The grasping and releasing tasks are initiated when the user applies a flexion force ( $F_{f}$ ) over the pre-set *Threshold*_*grasp*_ and *Threshold*_*release*_. A grasping task is achieved by rotating the motor from the initial angle, which was set when the cable was completely untwisted, to the holding angle when a grip strength ( $F_{g}$ ) reaches a 15 N. The releasing task is done by rotating the motor from the holding angle to the initial angle.

The orthosis comprises a passive elastic structure (including a beam) fabricated from thermoplastic polyurethane (TPU; Hyvision System, Inc. Seongnam, South Korea), an intention detection module, and a control module (Figure 2A). The orthosis has a total weight of 454 g, and it compactly fits on the affected hand and forearm. The passive elastic structure is made using a 3D printer (Cubicon Single, Hyvision System, Inc. Seongnam, South Korea) and reduces the number of actuators required by realizing the passive extension of fingers. The passive elastic structure allows independent donning/doffing of the orthosis and avoids joint compression during extension of spastic finger joints. The beam of the passive elastic structure where the fingers make contact is designed to optimize the stiffness for ensuring a sufficient grip aperture in the pre-grasping phase and minimizing the required antagonistic assistive force (*T*) for grasping. The passive elastic structure excluding the beam is designed as per the hand geometry of each user for comfort. Because of finger contracture in the affected hand, a 3D scanner (Sense2, 3D Systems Inc. Rock Hill, SC, United States) is used to scan the unaffected hand when the user grasps an 80-mm-diameter cylinder. The affected hand geometry is generated by mirroring the 3D scanning results assuming symmetry of the hands (Kulaksiz and Gözil, 2002). The thumb module is designed based on the affected hand geometry using the software Geomagic Freeform (3D Systems, Inc. Rock Hill, SC, United States) so that the palmar aspect of the thumb is rigidly fixed at the abducted posture as the user grasps an 80-mm-diameter cylinder.

The control module comprises a small motor (10 ∅ , 46.8 mm length, 64:1, 315172, MAXON Inc. Sachseln, Switzerland), cable (Power Pro, Shimano Inc. Caringbah, Australia), motor driver (446023, MAXON Inc. Sachseln, Switzerland), Arduino Micro, and battery (800 mAh-11.1v, PEAKPOWER Engineering Inc. Lakewood, United States). The cable contraction for active flexion of the beam is driven by a twisted string actuation (TSA) mechanism (Palli et al., 2013). The contraction length of the cable is adjusted by controlling the rotation angle ( φ ) of the motor. A contraction force (assistive force) is applied at routing structures of the beam. The battery continuously lasts for 170 min, and 2,260 cycles of active flexion were continuously achieved during that period. It was also confirmed that the orthosis could withstand ∼40,000 cycles of active flexion without break. The intention detection module comprises two force-sensing resistors (FSRs) (FSR01CE, OHMITE Manufacturing Co., Warrenville, IL, United States) that are respectively positioned on the beam and thumb module. In particular, the FSR on the beam (FSR_f_) can sense the flexion force of the affected hand, and the FSR on the thumb module (FSR_g_) can measure how much grip strength is applied to a grasped object.

Because stroke survivors cannot voluntarily extend their fingers because of extensor muscle weakness, the grasping and releasing intentions are detected solely using the FSR_f_ that can measure the flexion force. The grasping and releasing intentions are detected when the FSR_f_ detects a force that exceeds the pre-set values *Threshold*_*grasp*_ and *Threshold*_*release*_, respectively (Figure 2B). To calculate *Threshold*_*grasp*_ and *Threshold*_*release*_, the voluntary force of the user is first calculated during voluntary grasping in a setup procedure. The user applies the maximal flexion force for a grasping task while wearing the orthosis. The FSR_f_ measures the peak value for five attempts, and the values are then recorded. The baseline force, which corresponds to the force at rest, is measured before the voluntary grasping. The minimum difference between the peak and baseline forces is defined as the voluntary force. *Threshold*_*grasp*_ and *Threshold*_*release*_ were defined as the sum of the calculated voluntary force and baseline force. These values are updated at the end of every grasping and releasing task because the baseline force varies with spastic contractions over time. At the end of a grasping and releasing task, a waiting period of 2–4 s is implemented until the spastic response settles down, and the force measured after the waiting period is set as the baseline force for the next tasks. The waiting period is determined as per the time required for the force to settle down to 110% of the baseline force that is measured before the voluntary grasping during the setup procedure.

The grasping and releasing tasks are initiated when the user applies a flexion force (*F*_*f*_) that exceeds the pre-set values (*Threshold*_*grasp*_, *Threshold*_*release*_). In the initial state, the fingers are passively extended (open-hand posture) at the initial angle of the motor, which is set when the cable is completely untwisted. Active flexion of the beam is initiated once the user applies a force that exceeds *Threshold*_*grasp*_. The active flexion is driven by rotating the motor to twist the cable under a nominal step input current. The motor rotates until the angle limit, which is set to when the end of the beam makes contact with the thumb module. Once the rotation angle reaches the angle limit, a proportional–integral–derivative (PID) controller maintains the angle limit for safety. When the grip strength (*F*_*g*_) reaches 15 N (Yurkewich et al., 2019) before the motor rotates to the angle limit, the PID controller holds the rotation angle to maintain the holding angle that grasps objects at a 15-N grip strength. The minimally required grip strength of 15 N for grasping (Yurkewich et al., 2019) is used to increase the life cycle of the cable, which can break because of abrasion during twisting. To release an object, the user should flex the fingers to apply a force over *Threshold*_*release*_. Passive extension is achieved by reversely rotating the motor under a nominal input current, which untwists the cable. Once the rotation angle reaches the initial angle, the PID controller holds the initial angle. For false activation (i.e., unintended grasping and releasing) caused by physical perturbation, the user must reapply a force to return their hand to the desired posture.

### 2.2 Beam design

**FIGURE 3:**
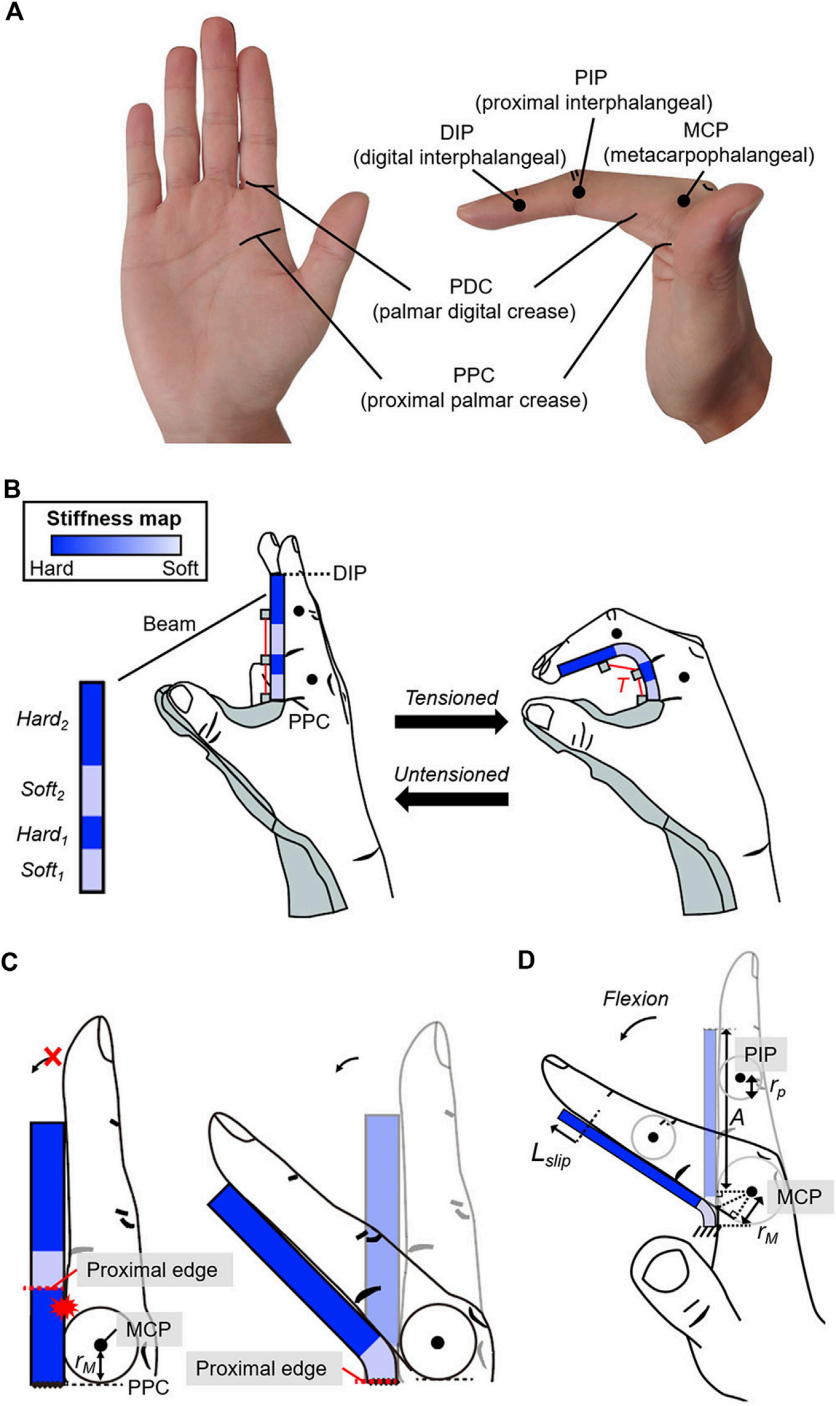
Geometric constraints of the beam to enable passive extension and active flexion of the fingers. **(A)** Hand landmarks used to design the beam. **(B)** The beam covers the palmar aspect from the PPC to the DIP to enable passive extension of the MCP and PIP joints. The stiffness near the MCP and PIP joints is set lower than that in other regions to allow active flexion of the joints under the assistive force ( T ). **(C)** The proximal edge of the soft region is set more proximal than the joint by a length greater than the radius of the joint to allow joint flexion. **(D)** Slip distance ( $L_{slip}$ ) considered for locating *Soft*_*2*_. *A* is the length between the MCP and DIP joints. *r*_*M*_ and *r*_*P*_ are respectively the radius of the MCP and PIP joints.

The beam geometry was designed to satisfy two conditions: it should passively extend the fingers in the pre-grasping phase, and it must be bendable to allow finger flexion under the action of the assistive force in the grasping phase. To achieve these conditions, the geometric constraints of the beam were determined based on the kinematics of the finger movement. The kinematics varies depending on the anthropometric dimensions of an individual hand. The positions of hand landmarks were used as a reference to measure the anthropometric dimensions and determine the geometric constraints on the beam design. The finger joints and following skin creases were selected as hand landmarks (Figure 3A): the palmar digital crease (PDC) and proximal palmar crease (PPC). Note that the PPC is where the proximal phalanx is folded toward the palm to achieve 90° metacarpophalangeal (MCP) flexion. To satisfy the first condition, the beam was designed to cover the proximal and middle phalanges of index and middle fingers (Figure 3B). This design allows the MCP and proximal interphalangeal (PIP) joints to be passively extended while sensory feedback is obtained *via* the fingertips.

To meet the second condition, the stiffness of the beam was designed as follows. Active flexion requires that the beam deflection along the MCP and PIP joints be significantly larger than in other regions; thus, stiffness close to the MCP and PIP joints should be less than that in other regions. The beam comprises two soft regions (*Soft*_*1*_ and *Soft*_*2*_) and two hard regions (*Hard*_*1*_ and *Hard*_*2*_) (Figure 3B). Because the beam is at the palmar aspect of the fingers, the proximal edge of soft regions was constrained to be more proximal than the corresponding joint by at least the radius of the joint to allow finger flexion (Figure 3C). The proximal edge of *Soft*_*1*_ was assigned to the PPC, which is more proximal than the MCP joint by the radius of the MCP joint (*r*_*M*_) for all users. The position of *Soft*_*2*_ was determined by considering the slip occurrence between the beam and fingers during MCP flexion. The beam slips with respect to the fingers (Figure 3D) during MCP flexion. Thus, the proximal edge of *Soft*_*2*_ was set to be more proximal than the PIP joint by a length that is greater than the sum (*L*_*const2*_) of the slip distance (*L*_*slip*_) and radius of the PIP joint (*r*_*p*_). Because 35° of MCP flexion has been reported to occur during tripod grasping (Park et al., 2014), *L*_*const2*_ that occurs during MCP flexion was used as a geometric constraint for *Soft*_*2*_, and it can be calculated as follows:

$$\begin{array}{l} L_{const2}=L_{slip}+r_{p}\\ \approx \left(A+r_{M}\tan\left(\frac{35^{\circ }}{2}\right)\right)\\ -\left(A-r_{M}\tan\left(\frac{35^{\circ }}{2}\right)\right)+r_{p}\\ =2r_{M}\tan\left(\frac{35^{\circ }}{2}\right)+r_{p} \end{array}$$ (1)

*A* (Figure 3D) is the length between the distal interphalangeal (DIP) and MCP joints at full extension. *r*_*M*_ and *r*_*P*_ are the radii of the MCP and PIP joints, respectively. The proximal edge of *Soft*_*2*_ was set to the PDC considered to be more proximal than the PIP joint by length greater than the *L*_*const2*_ (An et al., 1983; Buryanov and Kotiuk, 2010). Thus, the determined beam geometry comprises two soft regions (*Soft*_*1*_ and *Soft*_*2*_) and two hard regions (*Hard*_*1*_ and *Hard*_*2*_) at predefined locations to allow bidirectional finger movement.

**FIGURE 4:**
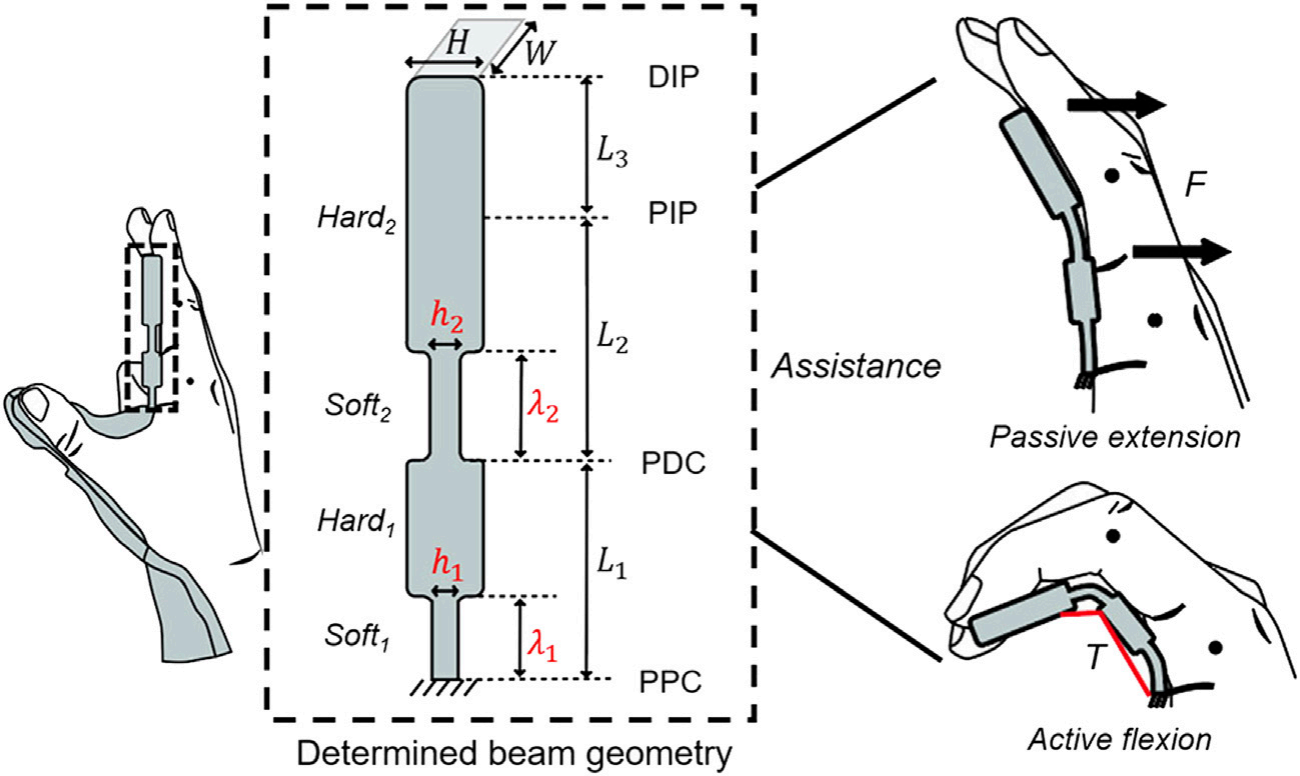
Determined beam geometry consisting of two soft regions (*Soft*_*1*_ and *Soft*_*2*_) and hard regions (*Hard*_*1*_ and *Hard*_*2*_) under the geometric constraints. The stiffness of the soft regions is determined by the design variables ( $h_{1},h_{2},\lambda_{1},\lambda_{2}$ ) calculated *via* stiffness optimization. The other variables are set when the anthropometric dimensions of individual hands are measured.

The stiffness can be adjusted by changing the thickness along the beam (Figure 4). The stiffness of *Soft*_*1*_ and *Soft*_*2*_ is determined by design variables ( $\lambda_{1},\lambda_{2},h_{1},h_{2}$ ), where $\lambda_{1},\lambda_{2}$ and $h_{1},h_{2}$ are the longitudinal length and thickness of the soft regions, respectively, and are determined *via* stiffness optimization to achieve sufficient grip aperture in the pre-grasping phase and minimal assistive force in the grasping phase. The other variables (*L*_*1*_, *L*_*2*_ *, L*_*3*_, *W*, and *H*) are determined as per the measured anthropometric dimensions of an individual hand. In particular, *L*_*1*_, *L*_*2*_, and *L*_*3*_ are the lengths between the PPC and PDC, PDC and PIP, and PIP and DIP, respectively. *W* is the width of the index and middle fingers. *H* is the thickness of the hard regions. To ensure high stiffness in the hard regions and the applicability of the Euler–Bernoulli theory, *H* is set so that the aspect ratio of the entire beam slightly exceeds 10. As geometric constraints, the distal edge of *Soft*_*1*_ should be more proximal than PDC ( $\lambda_{1}$ < *L*_*1*_), and the distal edge of *Soft*_*2*_ should be more proximal than PIP joint ( $\lambda_{2}$ < *L*_*2*_). The routing structures are positioned along the PPC, PDC, and PIP joint to deflect each of *Soft*_*1*_ and *Soft*_*2*_ under the assistive force.

### 2.3 Stiffness optimization

There exists a tradeoff between an achievable grip aperture and grip strength because an increase in the spatial stiffness leads to an increase in the grip aperture and required assistive force for grasping. The stiffness optimization enables the orthosis to achieve a target grip aperture in the pre-grasping phase while minimizing the required assistive force in the grasping phase. It would enable the orthosis to grasp common objects with increased grip strength by a given small-sized motor. Because stroke survivors have different joint characteristics (Kim et al., 2019) such as degree of spasticity, the stiffness should be optimized as per the measurements of individual hand parameters. Otherwise, the spatial stiffness distribution that is equally designed for all stroke survivors may result in a limited grip aperture for users with a severe degree of spasticity and or require an unnecessarily large amount of assistive force for users with flaccid hands. The stiffness optimization process has two steps: obtaining stiffness solutions that can achieve the target grip aperture and identifying the optimal solution that requires a minimal assistive force.

#### 2.3.1 Stiffness solutions for passive extension

Before determining the stiffness of *Soft*_*1*_ and *Soft*_*2*_, the relevant individual hand parameters should be measured. The anthropometric dimensions of the hand are the primary parameters. In this study, the dimensions were measured using a goniometer (Model 12-1012, Baseline^®^, Fabrication Enterprises, Inc. NY, United States), and they are used to determine *L*_*1*_, *L*_*2*_, *L*_*3*_, *W,* and *H* of the beam. Because finger contracture made it difficult to measure the affected hand, the dimensions were measured for the unaffected hand assuming the symmetry of both hands (Kulaksiz and Gözil, 2002). The joint angles that yield the target grip aperture of 80-mm (*ϕ*_*MCP*_ and *ϕ*_*PIP*_) are the secondary parameters. The target joint angles that should be maintained are used to determine the stiffness of *Soft*_*1*_ and *Soft*_*2*_. Because the joint angles differ among individuals because of the dissimilar hand size, the joint angles should be measured for each user and then be used to set constraints in determining the stiffness of *Soft*_*1*_ and *Soft*_*2*_. The joint angles were measured using the goniometer as a user grasps an 80-mm-diameter cylinder with their unaffected hand. The force applied to the beam by the fingers (i.e., joint resistive force) deflects *Soft*_*1*_ and *Soft*_*2*_ and decreases the grip aperture. This joint resistive force should be known to determine the necessary stiffness for *Soft*_*1*_ and *Soft*_*2*_ to achieve passive extension. Thus, the joint resistive force is the tertiary parameter. Because the joint resistive force differs depending on the degree of spasticity, it should be measured for each user.

**FIGURE 5:**
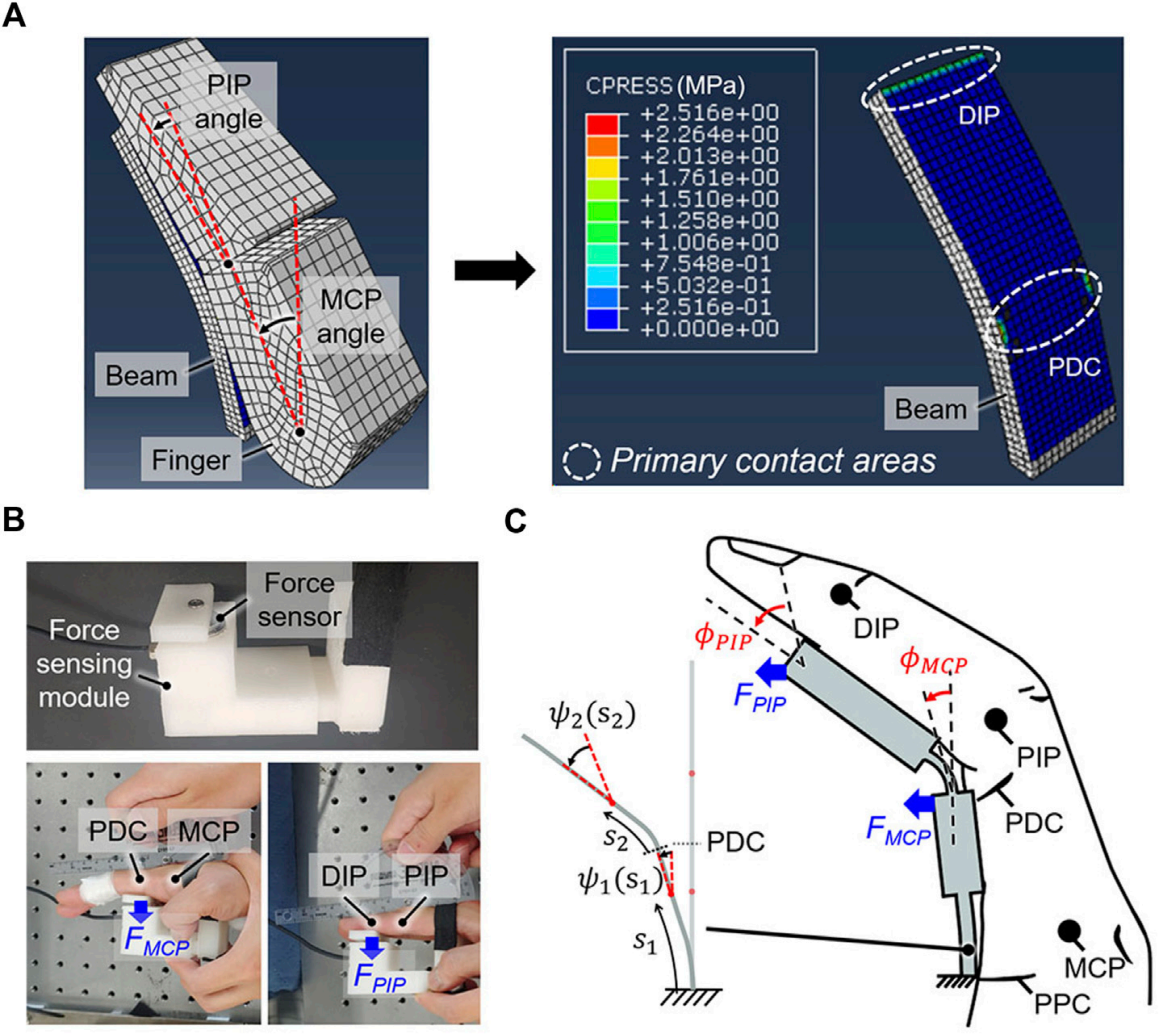
Obtaining the stiffness solutions of the beam to achieve passive extension. **(A)** The contact pressure is concentrated at the primary contact areas, which are located near the PDC and DIP when the finger is flexed against the beam to achieve a target grip aperture of 80 mm. **(B)** The joint resistive forces (*F*_*MCP*_, *F*_*PIP*_) at the primary contact areas, which are respectively applied to extend the MCP and PIP joints, were measured using a force sensor. **(C)** Calculating the stiffness solutions for which the beam deflection angles (*ψ*_1,_ *ψ*_2_) at the primary contact areas equal the individually measured joint angles ( $\phi_{MCP}$ ; $\phi_{PIP}$ ) for yielding an 80-mm grip aperture. The stiffness solutions were obtained based on the coordinates (*s*_*1*_, *s*_*2*_) of each beam segment, which were divided by the position of the PDC.

Before measuring the force, the location where the force is applied was obtained by static analysis under the condition that the finger and beam contact each other. In this study, finite element analysis (FEA) was used using ABAQUS (6.14, Dassault Systèmes Simulia Corp. Providence, RI, United States) for static analysis. An individual hand–beam model was developed (Figure 5A) that uses the measured dimensions of the index finger and position of the center of the joint rotation. The kinematics of the middle finger was assumed the same as that of the index finger for simplicity. The beam comprises two soft regions and two hard regions as per geometric constraints. Three longitudinal lengths (i.e., min, mid, and max) were used for each soft region of the beam. Because there is no thickness information for the beam, a constant thickness that satisfies the aspect ratio criterion was used for the entire beam. The hard regions were modeled to be rigid by applying kinematic constraints. A dynamic flexural modulus of 46.49 MPa was applied to the beam; this value was measured for TPU by operating a three-point bending test system (DTS Company, Menlo Park, CA, United States) at a strain rate of 0.1/s. The TPU was modeled as an elastic material because the TPU showed a linear stress-strain curve at low strain regions occurred during the active flexion. The modeled finger was flexed based on the individually measured joint angles, and it was found that the contact pressure is concentrated near the PDC and DIP joint (i.e., primary contact areas). Thus, the joint resistive forces were assumed to concentrate at positions of the PDC and DIP joint.

The joint resistive forces (*F*_*MCP*_ and *F*_*PIP*_) for the user were measured based on the positions of primary contact areas (Figure 5B). In this study, a force-sensing module containing a force sensor (KTOYO 247SA, KTOYO, Ltd. Uijeongbu, South Korea) was used for measurements. The user sits in a chair that is adjusted such that the base of the sternum is approximately parallel to the tabletop. The user then rests their affected arm on the table and extends the elbow as much as possible. The wrist is externally maintained in a neutral posture by the experimenter, and a force sensor at the position of the PDC (i.e., primary contact area) of the index finger to maintain fully extended MCP joint is used to measure *F*_*MCP*_. The PIP joints are taped to prevent fingertips from touching the force-sensing module. A force sensor at the position of the DIP joint (i.e., primary contact area) of the index finger to maintain fully extended PIP joint is used to measure *F*_*PIP*_. For this measurement, the experimenter externally holds the MCP joints in full extension. During the measurement, the force was nearly constant when the spastic response occurred right after the extension settled down. To eliminate the contribution of the spastic response immediately after movement, the force was measured for 1 min, and the average force over the last 10 s is considered as *F*_*MCP*_ or *F*_*PIP*_.

The measured hand parameters are used to obtain the stiffness of *Soft*_*1*_ and *Soft*_*2*_ such that the beam can maintain the required joint angles against the joint resistive forces. The beam can be divided into two segments as per the position of the PDC. Thereafter, *h*_*1*_ and *h*_*2*_ were calculated with respect to *λ*_*1*_ and *λ*_*2*_ as follows, respectively (Beléndez et al., 2005):

$$\begin{array}{l} \frac{d^{2}\psi_{1}}{ds_{1}^{2}}=\frac{12}{EWh{\left(s_{1}\right)}^{3}}\left(-\left(F_{MCP}+F_{PIP}\right)\cos\left(\psi_{1}\right)\right)\\ B.C_{1}:\psi_{1}\left(0\right)=0\\ \;\psi_{1}\left(L_{1}\right)=\phi_{MCP}\;and\;M_{1}\left(L_{1}\right)=M_{2}\left(0\right)\\ \frac{d^{2}\psi_{2}}{ds_{2}^{2}}=\frac{12}{EWh{\left(s_{2}\right)}^{3}}-(F_{PIP}\cos(\psi_{1}+\phi_{MCP}))\\ B.C_{2}:\psi_{2}\left(0\right)=0\\ \;\psi_{2}\left(L_{2}+L_{3}\right)=\phi_{PIP}\;and\;M_{2}\left(L_{2}+L_{3}\right)=0 \end{array}$$ (2)

where *ψ*_1_ (*s*_*1*_) and *ψ*_2_ (*s*_*2*_) are the beam deflection angles at *s*_*1*_ and *s*_*2*_, which are the arc lengths between the fixed end and a point on the corresponding segment (Figure 5C). *h* (*s*_*1*_) and *h* (*s*_*2*_) are the thickness at *s*_*1*_ and *s*_*2*_. *M*_*1*_, *M*_*2*_, and *E* are the corresponding bending moments and Young’s modulus. The beam stiffness should be set to ensure a grip aperture of 80 mm. The difference between the beam deflection angles and joint angles at the primary contact areas is < 2.95° on average (Figure 5A). Thus, the boundary conditions (B.C_1_ and B.C_2_) were set to make the beam deflection angles at the primary contact areas equal to the individually measured joint angles (*ϕ*_*MCP*_ and *ϕ*_*PIP*_). *Hard*_*1*_ and *Hard*_*2*_ were assumed to be sufficiently stiff to ensure that any changes in *ψ*_1_ (*s*_*1*_) and *ψ*_2_ (*s*_*2*_) are negligible. In this study, MATLAB (2018a, MathWorks Inc. Natick, MA, United States) was used to calculate *h*_1_ and *h*_2_ based on *λ*_*1*_ and *λ*_*2*_, which are pre-set to change in increments of 1 mm within the geometric constraints. Then, stiffness solutions that satisfy Eq. 2 can be obtained.

The Young’s modulus of TPU was measured under the condition of a quasi-static strain rate (0.0021/s) using a high-precision micromechanical test system (DTS Co., Menlo Park, CA, United States). This measured Young’s modulus (22.35 MPa) was used as *E* in Eq. 2 and associated with the target deflection required to maintain a grip aperture of 80 mm. In particular, the beam deflection was designed such that it would approach the target deflection over time because of creep. The creep-induced beam deflection was considered to ensure that the grip aperture asymptotically converged to 80 mm during the wearing period.

The spasticity of the flexor muscles can change over time and made it difficult to guarantee that the beam, which has fixed spatial stiffness distribution, would maintain the target grip aperture. A grip aperture less than the target of 80 mm would present a challenge in positioning common objects within the hand for grasping in the pre-grasping phase. Thus, a safety factor was applied to the degree of spatial stiffness; the spatial stiffness distribution was designed to achieve the target grip aperture against maximal joint resistive forces. The joint resistive forces are greatest when the fingers are fully extended. Thus, the maximal joint resistive forces statically measured at the fully extended posture (*F*_*MCP*_ and *F*_*PIP*_) were used in Eq. 2 to find the stiffness solutions.

#### 2.3.2 Optimizing the stiffness for active flexion

**FIGURE 6:**
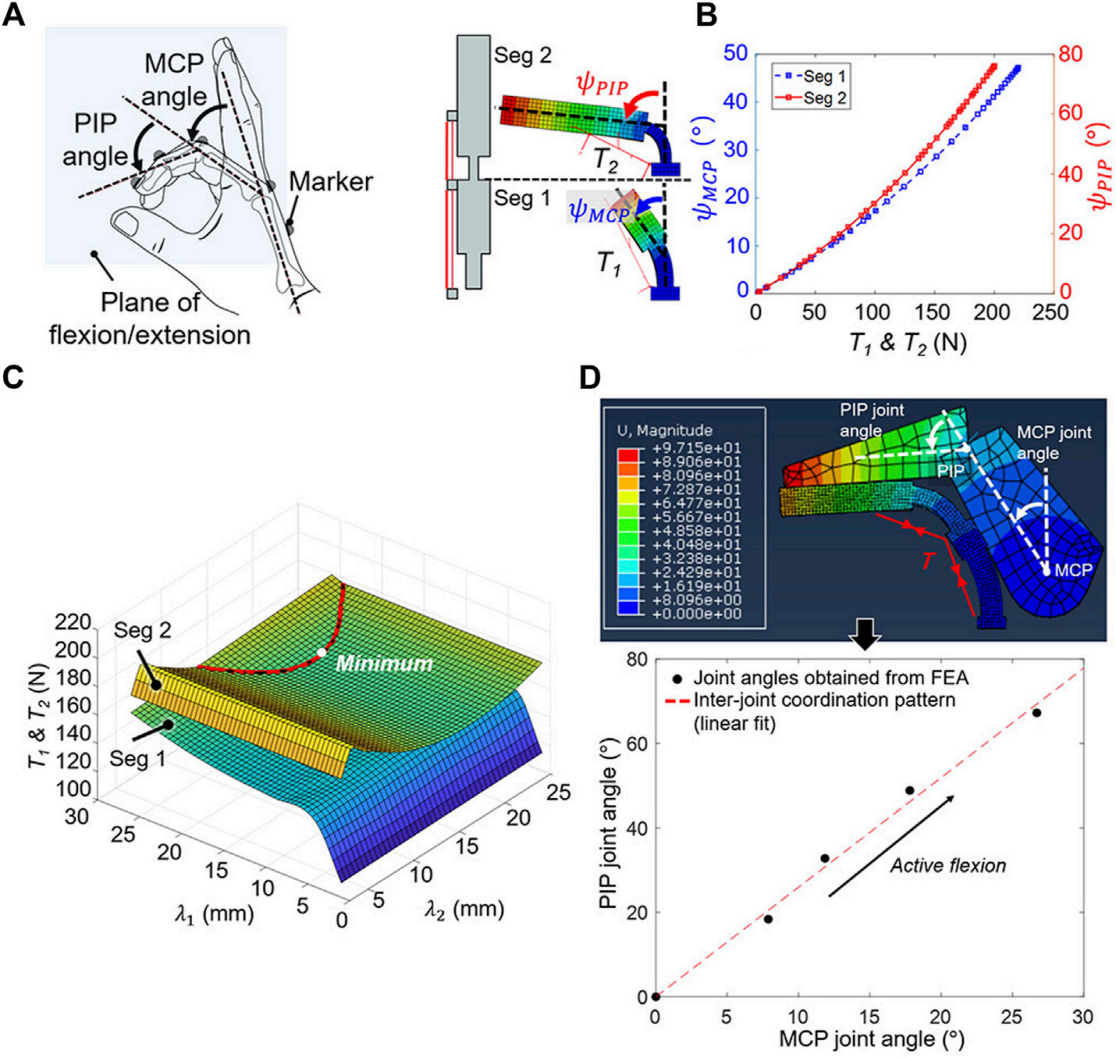
Finding the optimal solution among stiffness solutions that minimizes the assistive force for active flexion. **(A)** The MCP and PIP joint ROMs of the unaffected hand during tripod grasping were measured by capturing the 3D trajectories of the reflective markers. **(B)** The relationship between the assistive force ( $T_{1},T_{2}$ ) and beam deflection angle ( $\psi_{MCP},\psi_{PIP}$ ) was obtained *via* FEA of each beam segment (*Seg 1*, *Seg 2*) of the stiffness solutions, where the segment was divided by the position of the PDC. **(C)** The assistive force that yields the same beam deflection angles as the ROM measurements was calculated for the stiffness solutions. The optimal stiffness solution required minimal assistive force. $\lambda_{1}$ ; $\lambda_{2}$ represent the longitudinal lengths of the soft regions of the stiffness solutions. **(D)** An individual hand-beam model to obtain the inter-joint coordination pattern of joint angles during active flexion. The beam having the optimal stiffness solution was deflected under the assistive force (*T*), and finger joint angles when the finger makes contact with the beam were obtained.

To identify the optimal solution that requires a minimal assistive force for active flexion among the stiffness solutions, the amount of beam deflection required to emulate the tripod grasp (i.e., target grasp type) should be determined first. The ROMs of finger joints during a tripod grasp were used to determine the required beam deflection. The ROMs were measured during tripod grasping tasks using the unaffected hand (Figure 6A). In this study, the average results from three trials were recorded. Reflective markers were placed atop the fingertip, metacarpal, MCP, PIP, and DIP joints of the index finger to enable motion analysis with a motion capture system (OptiTrack V120: Trio, NaturalPoint, Inc. Corvallis, OR, United States). Principal component analysis was then applied to the 3D trajectory data of the reflective markers to identify the planes of flexion/extension movements. The 3D trajectory data was projected on the plane of flexion/extension, and the joint angles were calculated based on the projected trajectory data for relieving the influence of the abduction/adduction movement (Randazzo et al., 2017). Because the determined beam geometry allows finger flexion under beam deflection, the target beam deflection angles at hard regions were assumed the measured ROMs for assisting fingers with tripod grasping.

In this study, FEA was used to simulate the active flexion of stiffness solutions to obtain the relationship between beam deflection angles ( $\psi_{MCP},\psi_{PIP}$ ) and assistive forces (*T*_*1*_, *T*_*2*_) (Figure 6B). The beam as per the stiffness solutions was used for the simulations. Each beam was divided in two segments (*Seg 1* and *Seg 2*) as per the position of the PDC, where the routing structure was located. The relationships were obtained for each beam segment because the assistive force in *Seg 2* does not generate a moment in *Seg 1*. For the simulation, the assistive force was applied at the positions of the routing structures, and the beam segments were meshed with C3D20 elements and subjected to the dynamic flexural modulus (46.49 MPa). Plastic deformation resulting from active flexion was not considered because the plastic strain was expected to be small owing to the thinness of the beam.

The measured ROMs and relationships were used to determine the optimal solution that achieves the tripod grasp with the minimal assistive force. The ROM measurement results for the MCP and PIP joints were set as the targeted beam deflection angles of each segment for calculating *T*_*1*_ and *T*_*2*_. In particular, *T*_*1*_ and *T*_*2*_ that yield $\psi_{MCP}$ and $\psi_{PIP}$ corresponding to the ROMs were calculated for each stiffness solution based on the relationships shown in Figure 6B. The calculated *T*_*1*_ and *T*_*2*_ were then used to identify the optimal solution that required the minimal assistive force among the stiffness solutions (represented as *λ*_*1*_ and *λ*_*2*_ in Figure 6C).

The finger joint angles during active flexion were obtained using an individual hand–beam model. For the model, the optimal solution was used for the beam, and measured dimensions were used for the hand (Figure 6D). The beam was deflected under the assistive force (*T*), and finger joint angles when the finger makes contact with the beam were calculated. It was confirmed that the PIP joint angle increased linearly with respect to the MCP joint angle, and this inter-joint coordination pattern of the joint angles was obtained to check whether finger joint angles of users increase in accordance with the obtained inter-joint coordination pattern during assistance.

### 2.4 Experimental evaluation

#### 2.4.1 Participant recruitment

Ten stroke survivors having varying degrees of spasticity were recruited (Table 1) to evaluate the performance of the proposed orthosis at assisting with grasping tasks. The recruited participants were in the chronic stage with limited voluntary ROM. They could follow instructions and had no severe risk of skin breakdown for safety. The participants who scored a MAS grade of four were excluded because passive movement is physically difficult. Moreover, the participants with botulinum toxin type A injection were excluded because the injection can affect the degree of spasticity. The orthosis was personalized as per individuals and 3D-printed. Because the required assistive force for grasping increases as the stiffness of the orthosis increases, the motor that can actively flex the orthosis having the highest stiffness was selected and used for all orthoses to ease the design. The research was approved by the Institutional Review Board of KAIST (KH2020-59, 04/07/2020), and written consent was obtained from each participant after the experiment was explained.

**TABLE 1:** Stroke participant demographics and beam profiles.

Participant number	Months post- stroke	MAS	Aff/Dom hand	Gender	Age (yrs)	*F*_*MCP*_ (N)	*F*_*PIP*_ (N)	*h*_*1*_ */λ*_*1*_ (mm/mm)	*h*_*2*_ */λ*_*2*_ (mm/mm)	*W* (mm)
P1	95	2	R/R	F	52	1.55	0.88	3.45/16.0	2.73/12.3	40
P2	568	0	L/R	F	67	0.33	0.16	1.54/21.0	1.11/12.5	42
P3	237	0	L/R	F	69	0.28	0.47	2.23/15.4	1.64/8.9	40
P4	288	1+	R/R	M	61	0.84	1.33	2.66/7.2	3.25/14.1	43
P5	86	1	R/R	M	59	0.17	0.84	2.60/17.0	1.74/10.3	42
P6	131	2	L/R	M	63	0.54	1.79	2.97/10.6	2.66/17.8	40
P7	146	1+	L/R	F	61	0.73	0.73	2.82/18.0	1.50/4.0	38
P8	109	1	R/R	F	66	0.18	0.39	2.10/19.0	1.16/5.3	35
P9	104	1+	L/R	F	77	0.99	0.93	3.11/21.0	1.64/6.3	40
P10	69	3	L/R	M	70	4.62	6.95	5.95/19.9	4.71/16.8	43

Ten chronic stroke participants with different hand anthropometric dimensions and degrees of spasticity were recruited.

*F_MCP_, F_PIP_, denote joint resistive forces and h_1_, h_2_, λ_1_, λ_2_, W denote dimensions of the beam (thickness, longitudinal length, and width).

#### 2.4.2 Evaluation criteria

The following criteria were used to evaluate the performance of the proposed orthosis design.

**FIGURE 7:**
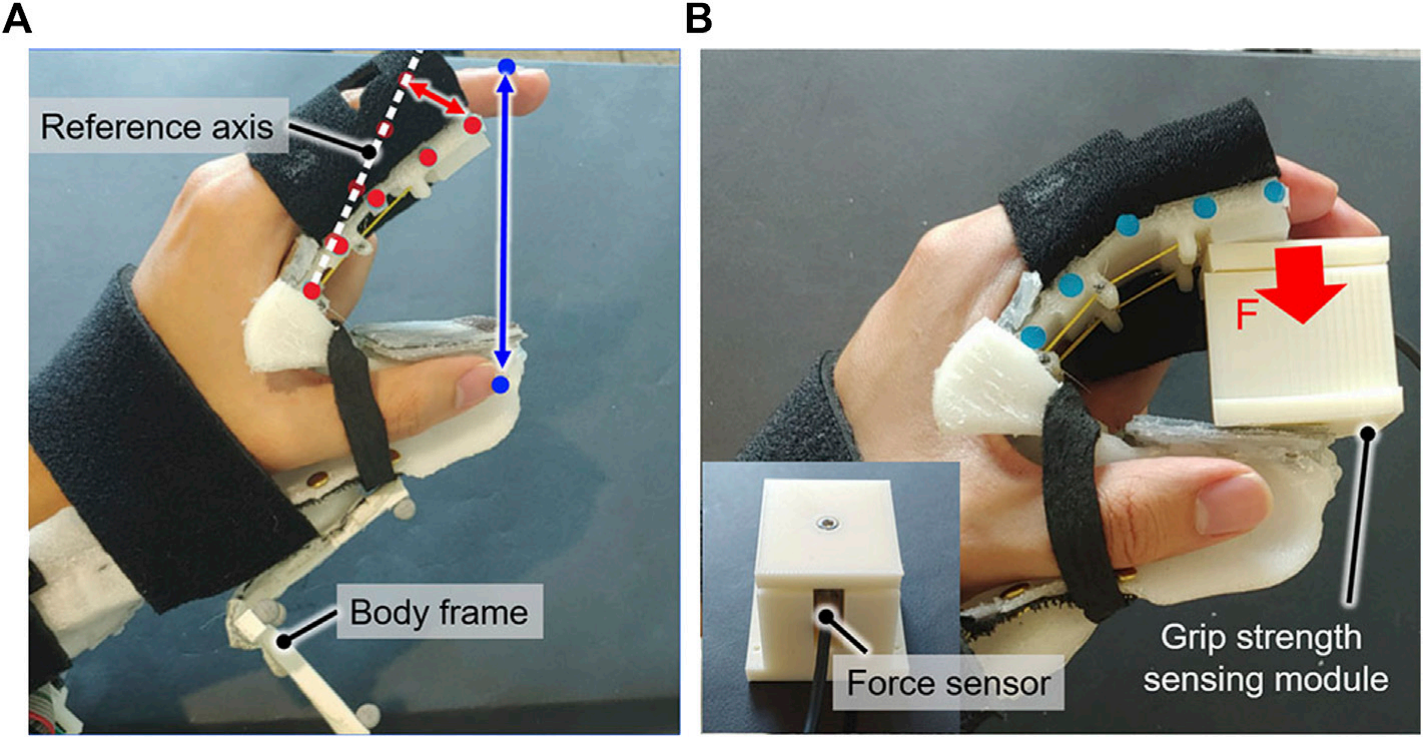
Experimental setup for evaluating the orthosis. **(A)** The beam deflection at the tip (red arrow) and grip aperture (blue arrow) were measured by tracking reflective markers, which were attached to the upward-facing side of the beam (red dot) and fingertips (blue dot). The beam deflection was measured with respect to the reference axis, which was determined based on the markers prior to the orthosis being worn. **(B)** The grip strength of the orthosis was measured using a grip strength–sensing module under an applied assistive force.

1) Beam deflection and grip aperture: The beam deflection was measured to confirm whether it was equal to the target deflection. The target deflection was obtained using FEA by applying the maximal joint resistive force to the beam having the measured Young’s modulus. The increase in the deflection of the distal end of the beam was used (Figure 7A) to determine whether the beam is deflected closed to the target deflection to yield the target grip aperture. It was measured immediately after being donned by tracking five reflective markers that were positioned along the upward-facing side of the beam. Because it has been reported that no significant plastic deformation occurs after four cycles of active flexion (Qi and Boyce, 2005), the beam deflection was measured again after four repetitions of active flexion. The grip aperture was measured based on markers attached to the index fingertip and thumb tip to verify whether it achieved the target grip aperture of 80 mm.
2) ROM measurement: The ROMs for the MCP and PIP joints of the index finger were measured before and after the orthosis was applied to the affected hand. Reflective markers were placed atop the metacarpal, MCP, PIP, and DIP joints and the fingertip of the index finger for tracking, and joint angles were obtained based on the 3D trajectory data of the markers. The voluntary ROM (i.e., ${ROM}_{voluntary}$ ), for which participants flexed their fingers to achieve a closed-hand posture from the maximal extended posture, was calculated from a single trial. The assisted ROM (i.e., ${ROM}_{assisted}$ ) was calculated from a single trial when the beam was deflected until contact was made with the thumb module. The increase in ROM (i.e., $\Delta {ROM}_{total}$ ) was calculated as follows:

$$\begin{array}{l} \Delta ROM_{total}=\Delta ROM_{MCP}+\Delta ROM_{PIP}\\ =ROM_{assisted}-ROM_{voluntary}\\ \;\Delta \theta =\theta_{voluntary}-\theta_{assisted} \end{array}$$ (3)

where ${\Delta ROM}_{MCP}$ and ${\Delta ROM}_{PIP}$ are the increases in the ROMs of the MCP and PIP joints, respectively. The increase in joint extension (i.e., *∆θ*) was obtained, where *θ*_*voluntary*_ and *θ*_*assisted*_ are the maximal unassisted and assisted extension angles, respectively.

3) Grasp performance: The maximum grip strength with the orthosis, in addition to the ability of the orthosis to grasp various objects, were evaluated. The objects applied for this test were selected based on the previous study (Kang et al., 2016), and they were as follows: a glass cup, baseball, stapler, golf ball, banana, and rectangular battery. The maximum grip strength was measured at the angle limit of the motor when the participant grasped a rectangular 40-mm-height (i.e., 50% of the target grip aperture) grip strength–sensing module without any voluntary force. For this measurement, the motor was rotated until the angle limit regardless of the grip strength measured by the FSR sensor. The force sensor (KTOYO 247SA, KTOYO, Ltd. Uijeongbu, South Korea) was embedded in the grip strength–sensing module (Figure 7B).
4) Functional assessment and questionnaire: Each participant was instructed to perform nine ADL tasks from the Chedoke Arm and Hand Activity Inventory (CAHAI)-9 (Barreca et al., 2004) to evaluate the assistive performance of the orthosis with grasping tasks. All nine tasks were bimanual activities: 1) opening a jar of coffee, 2) dialing an emergency number, 3) drawing a line with a ruler, 4) pouring a glass of water, 5) wringing out a washcloth, 6) fastening five buttons, 7) drying the back with a towel, 8) putting toothpaste on a toothbrush, and 9) cutting medium-resistance putty. The performance of each ADL task was evaluated on a scale of 1–7 by the experimenter according to the CAHAI-9 criteria (Jane Rowland et al., 2011). Both hands were positioned on the table, and the participants were instructed to refrain from placing their elbows on the table. Because the participants lacked fine control of the affected arm, it was supported by a gravity compensator and experimenter. The participants first performed all nine tasks without the orthosis. After a 5 min break, they repeated tasks with the orthosis under the same environmental conditions. The performance improvement was quantitatively evaluated by comparing the scores with and without the orthosis. After conducting the functional assessment, the participants were asked to answer a questionnaire comprising 1) the weight of the orthosis is manageable, 2) the assistance of the orthosis is satisfactory, and 3) the orthosis is comfortable while using. The questions were determined based on the orthotics and prosthetics user’s survey (OPUS) (Heinemann et al., 2003). The satisfaction of the participants was ranked from one to five, in which the value increases with higher satisfaction.

## 3 Results

### 3.1 Beam deflection and grip aperture

The beam deflection was less than the target deflection, thereby yielding a grip aperture larger than the target grip aperture of 80 mm. The distal end of the beam deflected by 12.17 ± 10.00 mm immediately after donning, generating the grip aperture of 94.26 ± 10.73 mm. This measured deflection was less than the target deflection of 40.08 ± 7.37 mm primarily owing to viscosity of the TPU. Because of creep occurred after donning and plastic deformation by active flexion, the beam deflection increased by 9.38 ± 5.48 mm after four repetitions of active flexion, which corresponded to an average decrease of 6.74 mm for the grip aperture. Non-etheless, the measured beam deflection of 21.54 ± 9.36 mm still did not equal to the target deflection of 40.08 ± 7.37 mm, yielding a grip aperture of 87.52 ± 6.40 mm. The error for the beam deflection would be because of the safety factor considered in designing the stiffness of the beam. The beam stiffness was set to yield the target grip aperture against the maximal joint resistive force; however, fingers would apply force less than the maximal joint resistive force on the beam. This would primarily make the measured deflection be less than the target deflection, which led to increase in the grip aperture.

### 3.2 ROM measurement

**FIGURE 8:**
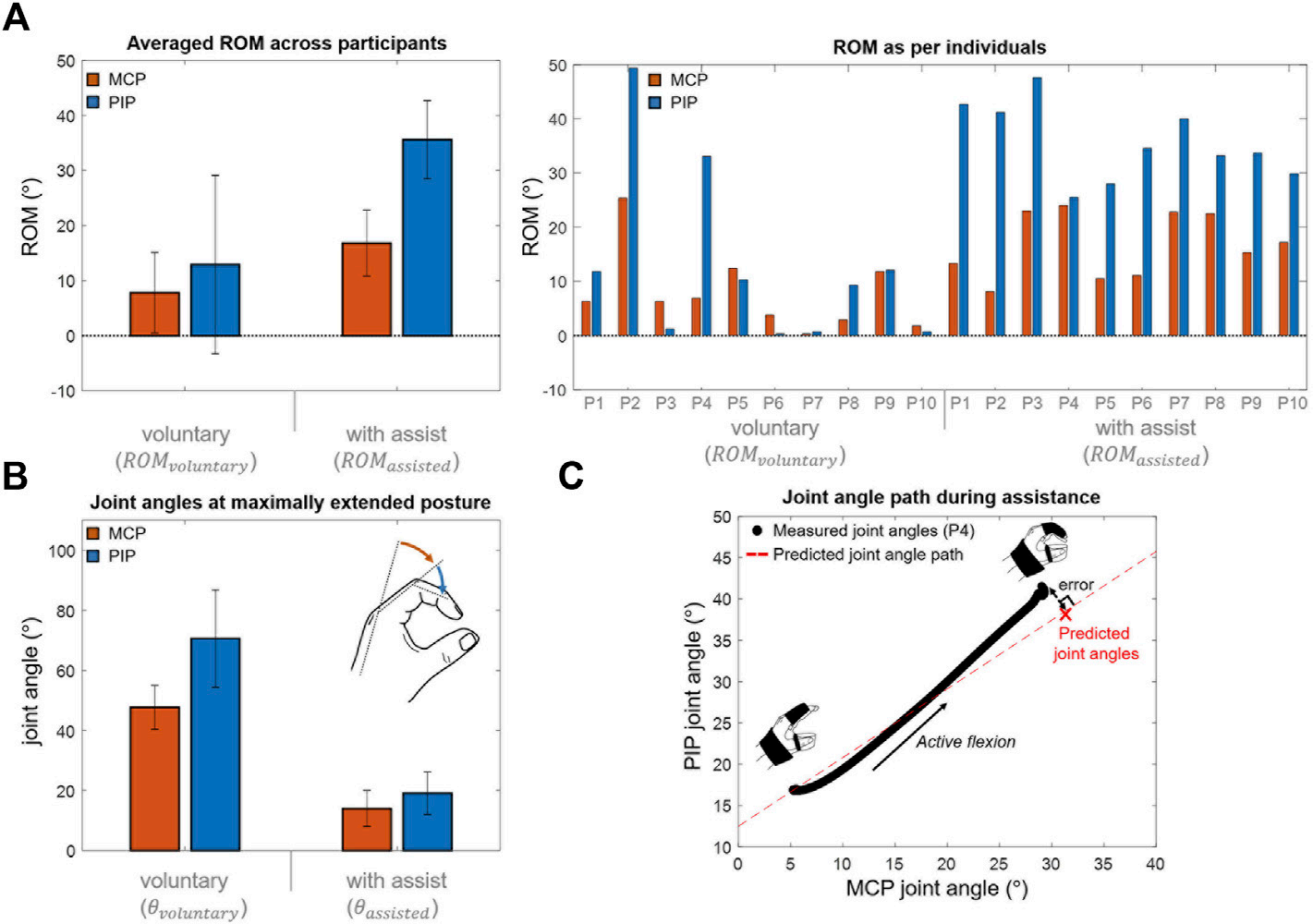
Experimental results. **(A)** Averaged ROM across participants during voluntary and assisted flexions (mean difference of MCP and PIP joint ROMs = 9.0 ° , 22.8 ° ; paired t-test, *p* = 0.0427, 0.0033) and the ROM of individuals. **(B)** Averaged joint angles across participants at maximally extended posture without and with assistance (mean difference of MCP and PIP joint angles = 33.6 ° , 51.4 ° ; paired t-test, *p* = 2.1e-5, 3.1e-5). **(C)** The measured joint angle path during active flexion. The finger joint angles increased in accordance with the inter-joint coordination pattern obtained from the individual hand-beam model. The “Measured joint angles” was obtained from 3D trajectories of markers attached on the finger. The “Predicted joint angle path” was obtained by increasing joint angles from the maximal assisted extension posture according to the calculated inter-joint coordination pattern.

The orthosis effectively assisted the finger joints for all participants (Figures 8A, B). All participants demonstrated a limited voluntary ROM of 20.7° on an average because of spastic finger joints. They were only able to flex their fingers from a neutral posture to a closed-hand posture. Although the orthosis-enabled finger flexion was limited by the thumb module, the orthosis increased the joint ROM by 31.7° ± 26.6°. Note that the finger flexion was not restricted by the thumb when the voluntary ROM was measured. The ROMs of the MCP and PIP joints were increased by 9.0° ± 12.1° and 22.8° ± 18.2°, respectively. Moreover, the orthosis provided passive assistance to yield a maximal assisted extension angle of 33.2° ± 12.0°; the fingers were significantly more extended compared to the maximal voluntary extension (maximal unassisted extension angle of 118.2° ± 25.8°).

The finger joint angles increase in accordance with the inter-joint coordination pattern obtained by the individual hand–beam model. The “Measured joint angles” in Figure 8C was obtained based on the measurement results during active flexion. The “Predicted joint angle path” in Figure 8C was obtained by increasing joint angles from the maximal assisted extension posture according to the inter-joint coordination pattern. It was confirmed that the joint angles increase in accordance with the inter-joint coordination pattern. The error between the joint angles at maximal flexed posture and predicted joint angles were 4.9° ± 2.9° which were respectively 4.3° ± 2.6° in the MCP joint and 2.2° ± 1.4° in the PIP joint. The joint resistive force was not considered in the hand-beam model to obtain the inter-joint coordination pattern, and it would result in the error which was only 9.3% of assisted joint ROM on average. The inter-joint coordination of the MCP and PIP joints during assistance can be controlled by spatial stiffness distribution design.

### 3.3 Grasp performance

All participants could grasp each object with the orthosis. Furthermore, they were able to establish contact between their fingertips and large-size objects (i.e., the glass cup and baseball). However, the large slip between the fingers and beam prevented the fingertips from contacting the small-size objects (i.e., banana, stapler, golf ball, and battery). The maximum grip strength was measured as 26.27 ± 3.90 N, and the orthosis showed a high grip strength-to-weight ratio (Table 2).

**TABLE 2:** Characteristics of active hand orthoses.

Device	Actuators	Number of actuators	Number of assisted fingers	Weight (orthosis/total)	Maximum grip strength	Maximum grip strength/Total weight
Hand of Hope (Ho et al., 2011)	motors, cable-driven	5	5	700 g/n/a	12 N (pinch)	n/a
Yap et al., 2018	pneumatic, fabric-reinforced	5	5	180 g/1260 g	36.2 N (power)	0.029 N/g
Nycz et al., 2016	motors, cable-driven	4	4	113 g/867 g	8.7 N (pinch)	0.01 N/g
Graspy-Glove (Popov et al., 2017)	motors, cable-driven	4	4	250g/340 g	16 N (pinch)	0.047 N/g
Ryu et al., 2008	hydraulics, cable-driven	3	3	2,620 g/2,740 g	12 N (pinch)	0.004 N/g
SNU Exo-Glove (In et al., 2015)	motors, cable-driven	3	3	194 g/n/a	20 N (pinch)	n/a
HERO grip glove (Yurkewich et al., 2020)	motor, cable-driven	2	5	n/a/284 g	11.0 N (pinch)	0.039 N/g
HERO glove (Yurkewich et al., 2019)	motor, cable-driven	1	5	n/a/192 g	0 N (pinch)	0 N/g
**Proposed orthosis (this paper)**	**motor, cable-driven**	**1**	**3**	**259g/454 g**	**26.27 N (pinch)**	**0.058 N/g**

### 3.4 Functional assessment and questionnaire

**FIGURE 9:**
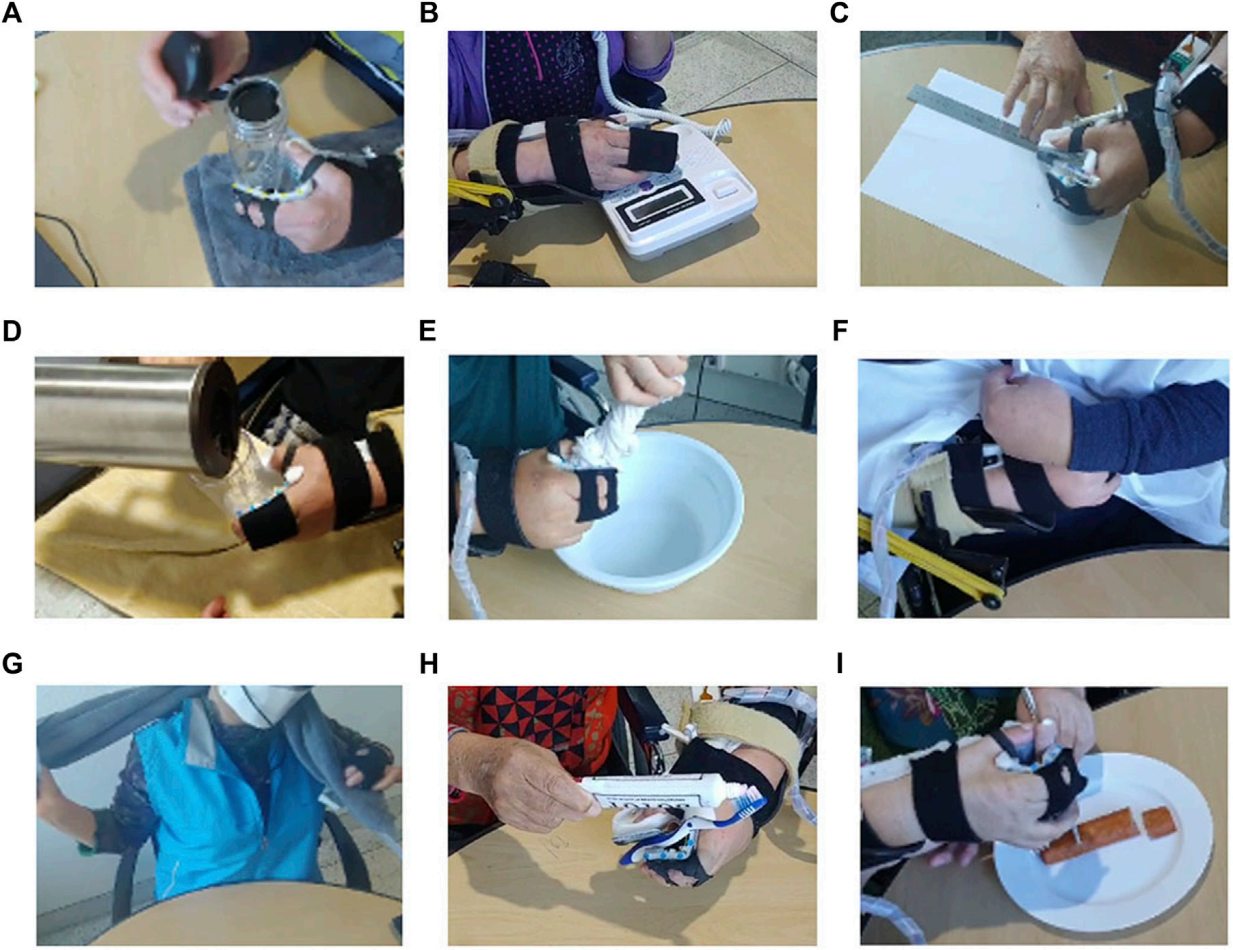
Tasks 1–9 from the Chedoke Arm Hand Activity Inventory (CAHAI)-9. **(A)** Task 1: Opening a jar of coffee. **(B)** Task 2: Dialing an emergency number. **(C)** Task 3: Drawing a line with a ruler. **(D)** Task 4: Pouring a glass of water. **(E)** Task 5: Wringing out a washcloth. **(F)** Task 6: fastening five buttons, **(G)** Task 7: Drying the back with a towel. **(H)** Task 8: Putting toothpaste on a toothbrush. **(I)** Task 9: Cutting medium-resistance putty.

The results of the CAHAI-9 tasks (Table 3) demonstrated that all participants had difficulty without the orthosis owing to contracture and low grip strength. The score of one or two was assigned for all tasks because participants do not voluntarily show any manipulation of the affected hand. With orthosis, all participants were able to complete the tasks except for tasks 2, 6, and 9 with only arm support and cueing (Figure 9). The orthosis increased the grip aperture and grip strength of participants to improve their performance of most tasks (score increase: 14.40 ± 1.78). To precisely locate targeted objects along the beam for grasping, the unaffected hand was used for positioning because the orthosis cannot assist with in-hand and upper-limb manipulations. Non-etheless, with only the passive assistance of the arm, the orthosis enabled the participants to independently accomplish bimanual tasks by stably holding targeted objects with the affected hand. Specifically, for task 1, they could open a lid of the jar using the unaffected hand because the affected hand could hold a jar. For task 3, they could draw a line by grasping a pen with the affected hand while the unaffected hand is fixing the ruler. For task 4, participants held a glass cup using the affected hand while pouring water with the unaffected hand. For task 5, one side of a washcloth was held by the affected hand, and the unaffected hand wrung out a wash cloth. For task 7, one side of a towel was held by the affected hand, and the unaffected hand dried the back with a towel. Thus, stroke survivors were assigned score of four for these tasks because they can accomplish 100% of tasks with arm support. For task 8, although it was possible to hold a toothpaste using the affected hand to make the unaffected hand unscrew a lid, squeezing the toothpaste using only the affected hand was impossible with the control algorithm used for the orthosis. Thus, a score of three was assigned for task eight by considering that 50% of the task is accomplished. The orthosis could not provide adequate assistance in tasks 6 and 9 because the tripod grasp type was insufficient to manipulate the object. Task two was not completed because the participants were unable to prevent their ring and little fingers from pushing non-targeted buttons.

**TABLE 3:** Task-based functional assessment results.

Participant number	Task 1	Task 2	Task 3	Task 4	Task 5	Task 6	Task 7	Task 8	Task 9	Total score	Performance improvement
P1	2/4	1/1	2/4	1/4	2/4	1/1	2/4	2/3	2/1	15/26	11
P2	1/4	1/1	1/4	1/4	2/4	1/1	1/4	2/3	1/1	11/26	15
P3	1/4	1/1	1/4	1/4	1/4	1/1	1/4	2/3	1/1	10/26	16
P4	2/4	1/1	1/4	2/4	2/4	1/1	1/4	2/3	1/1	13/26	13
P5	1/4	1/1	2/4	1/4	2/4	1/1	2/4	2/3	1/1	13/26	13
P6	1/4	1/1	2/4	1/4	2/4	1/1	1/4	2/3	1/1	12/26	14
P7	1/4	1/1	1/4	1/4	1/4	1/1	1/4	1/3	1/1	9/26	17
P8	1/4	1/1	1/4	1/4	1/4	1/1	1/4	2/3	1/1	10/26	16
P9	1/4	1/1	2/4	1/4	2/4	1/1	1/4	2/3	1/1	12/26	14
P10	1/4	1/1	2/4	1/4	2/4	1/2	1/4	2/3	1/1	12/27	15
Mean (SD)											14.40 (1.78) *p* = 1.01e-09

ADL, performance (without orthosis/with orthosis) was quantitatively scored based on the criteria for the CAHAI-9, clinical test. Task 1-9 are respectively opening a jar of coffee, dialing an emergency number, drawing a line with a ruler, pouring a glass of water, wringing out a washcloth, fastening five buttons, drying the back with a towel, putting toothpaste on a toothbrush, and cutting medium-resistance putty. The *p*-value was calculated using a paired t-test.

The participants answered that the device is sufficiently lightweight (score: 4.3 ± 0.7) and comfortable (score: 4.2 ± 0.5). Moreover, they were satisfied with the assistance of the orthosis (score: 4.3 ± 0.48).

## 4 Discussion

In this study, a personalized spatial stiffness distribution is presented for a portable and strengthful hand orthosis. The stiffness was optimized for each participant based on measurement of individual hand parameter to satisfy predetermined requirements for the orthosis: maintaining a minimum grip aperture of 80 mm and minimizing the assistive force needed to achieve flexion. The stiffness optimization of the passive elastic structure assists individuals with finger flexor spasticity to achieve the target grip aperture to grasp common objects while minimizing the antagonistic assistive force to generate sufficient grip strength. The experimental results demonstrated that the orthosis achieves a high grip strength-to-weight ratio and weighs only 454 g, including all of the motor, electronics, and battery located at the hand and forearm. It was demonstrated that the strengthful all-in-one orthosis achieved by the spatial stiffness distribution design can assist participants with the bidirectional movement of the affected hand to grasp various objects and thus can aid with bimanual tasks for ADL.

The passive elastic structure enabled spastic finger joints to be extended even for a participant who has MAS grade of three. Previous orthoses (Lee et al., 2014; Nycz et al., 2016; Popov et al., 2017; Kim and Park, 2018; Yurkewich et al., 2020) assist with extension using cables as exotendons. However, for stroke survivors with a severe degree of spasticity, the tension applied by exotendons to assist with extension may apply a relatively large compressive force on the finger joints, which is a safety concern (Felson, 2013; Kim et al., 2019). The proposed orthosis includes a passive elastic structure that is positioned on the palmar aspect of the affected hand to eliminate the requirement for exotendons and increase the moment arms of the extending forces applied at finger joints. Participants replied that they did not feel any pain and discomfort during assisted finger joint extension. The passive elastic structure would enabled the orthosis to assist finger extension against spastic finger joints with reduced joint compression.

The spatial stiffness distribution design of the orthosis significantly affects ROMs of finger joints during assistance. In this study, by adjusting the stiffness along the beam considering the slip between the fingers and beam, the limited increase in ROM during assistance is avoided. In particular, the hard region of beam can limit the ROM of the PIP joint when the slip during finger flexion is not considered in the spatial stiffness distribution design. The soft regions were respectively positioned close to the finger joints considering the slip, and their stiffness were adjusted to emulate the tripod grasp. The proposed design prevents the beam from blocking finger flexion and enables users to conduct tripod grasp. The proposed orthosis was demonstrated to increase the ROM of finger joints by an average of 31.7°, which is similar to that of a previous orthosis (i.e., 46.3°) (Yurkewich et al., 2019).

The results highlight the importance of a user-specific orthosis design. To maximize user compliance and increase grip strength through reduced actuator size and weight, orthosis was personalized for each participant based on the measurements of hand parameters. This was achieved by first considering the individual hand geometry. With the exception of the beam component, the passive elastic structure was designed based on the affected hand geometry generated by 3D scanning. All participants reported that the orthosis was comfortable. The second individualized characteristic that was considered was the joint resistive force, particularly for MCP and PIP joints. The spatial stiffness distribution was personalized based on measurement results of joint resistive force to ensure sufficient grip aperture and high enough grip strength, and it was shown that the orthosis can achieve grasping assistance with a high grip strength-to-weight ratio. The required motor size and weight for grasping assistance would be reduced by considering individual joint characteristics in the spatial stiffness distribution design.

The TSA mechanism was selected to improve the portability of the orthosis using its high contraction force-to-torque ratio and mechanical simplicity. Owing to its characteristics, the actuating module adopting the TSA mechanism becomes compact in comparison with the other actuating modules such as soft fluidic actuators (Zhang et al., 2019). However, the TSA mechanism has the disadvantage of contraction length. It can maximally generate contraction length as 30% of its untwisted length, and this should be considered in hardware design. In this study, the motor was mounted at the forearm to provide the untwisted length required for generating contraction length to flex the orthosis.

The proposed orthosis does have limitations. First, the cable of the TSA mechanism can break during use. The cable breaks after being twisted several times (Palli et al., 2013), which may decrease the usability. Devising an easily replaceable cable module may solve this limitation. Second, the orthosis was unable to assist with two tasks from CAHAI-9 (i.e., cutting medium-resistance putty and fastening five buttons). This is because the single tripod grasp was insufficient to perform these tasks. In particular, the limited degrees of freedom of the orthosis ultimately hindered manipulation of the objects, which prevented it from assisting the participants with tasks requiring dexterous manipulation. For the putty-cutting task, the applied torque caused the fork to rotate when the participant attempted to pierce the putty. For button fastening, dexterous manipulation was required to pull holes in the cloth toward the buttons.

In this study, a personalized spatial stiffness distribution design is presented for a portable and strengthful hand orthosis to assist with grasping tasks. The objective was to design spatial stiffness distribution that can assist bidirectional finger movement with a sufficient grip aperture in the pre-grasping phase and high enough grip strength in the grasping phase. The orthosis includes a passive elastic structure with an individually optimized stiffness and a cable contracted by TSA mechanism using a single motor. In the experiment, the orthosis can assist with grasping tasks for ADL by providing a sufficient grip aperture and grip strength. The orthosis increased the grip aperture and grip strength of all participants, enabling successful grasping even by those with severe degrees of spasticity.

## Data Availability

The raw data supporting the conclusions of this article will be made available by the authors, without undue reservation.
